# FOXO1 promotes cancer cell growth through MDM2-mediated p53 degradation

**DOI:** 10.1016/j.jbc.2024.107209

**Published:** 2024-03-21

**Authors:** Haruki Tomiyasu, Makoto Habara, Shunsuke Hanaki, Yuki Sato, Yosei Miki, Midori Shimada

**Affiliations:** 1 Department of Veterinary Biochemistry, Yamaguchi University, Yamaguchi, Yamaguchi, Japan; 2 Department of Molecular Biology, Nagoya University, Graduate School of Medicine, Showa-ku, Nagoya, Japan

**Keywords:** FOXO1, protein degradation, calcineurin, p53, MDM2

## Abstract

FOXO1 is a transcription factor and potential tumor suppressor that is negatively regulated downstream of PI3K-PKB/AKT signaling. Paradoxically, FOXO also promotes tumor growth, but the detailed mechanisms behind this role of FOXO are not fully understood. In this study, we revealed a molecular cascade by which the Thr24 residue of FOXO1 is phosphorylated by AKT and is dephosphorylated by calcineurin, which is a Ca^2+^-dependent protein phosphatase. Curiously, single nucleotide somatic mutations of FOXO1 in cancer occur frequently at and near Thr24. Using a calcineurin inhibitor and shRNA directed against calcineurin, we revealed that calcineurin-mediated dephosphorylation of Thr24 regulates FOXO1 protein stability. We also found that FOXO1 binds to the promoter region of MDM2 and activates transcription, which in turn promotes MDM2-mediated ubiquitination and degradation of p53. FOXO3a and FOXO4 are shown to control p53 activity; however, the significance of FOXO1 in p53 regulation remains largely unknown. Supporting this notion, FOXO1 depletion increased p53 and p21 protein levels in association with the inhibition of cell proliferation. Taken together, these results indicate that FOXO1 is stabilized by calcineurin-mediated dephosphorylation and that FOXO1 supports cancer cell proliferation by promoting *MDM2* transcription and subsequent p53 degradation.

FOXO1 is a member of the Forkhead box O (FOXO) family of transcription factors that are negatively regulated downstream of PI3K–AKT signaling (1). FOXO1 serves as a critical regulator of glucose metabolism by controlling the expression of genes related to gluconeogenesis in the liver and influencing pancreatic β-cell function (2, 3). FOXO1 is also considered a tumor suppressor because it regulates the transcription of genes that play important roles in cell cycle arrest and apoptosis induction (4, 5, 6, 7). Paradoxically, several studies have reported that FOXO1 supports tumor growth.

The first reported function of link between mammalian FOXO and cancer was that it forms a fusion protein with an oncogenic protein. FOXO-related chromosomal translocations give rise to FOXO1-PAX3/7, FOXO3-MLL, or FOXO4-MLL fusion proteins in alveolar rhabdomyosarcoma and acute leukemia (8, 9, 10). In addition, single-nucleotide somatic mutations in FOXO1, leading to over-activation of FOXO1, are frequently found in follicular lymphomas and diffuse large B-cell lymphomas (11, 12, 13). In gastric cancer, S256-phosphorylated FOXO1 correlates with higher overall survival and lower tumor angiogenesis, suggesting that activated FOXO1 supports tumor growth and metastasis (14, 15).

High expression of FOXO3 is associated with decreased overall and relapse-free survival in patients with acute myeloid leukemia (16). Studies focusing on FOXO3 localization in breast and colorectal cancers have shown that higher levels of FOXO3 nuclear localization correlate with lower overall survival (17, 18). In addition, FOXO4 has been found to be upregulated in response to doxorubicin and phenylbutyrate treatment in B cell lymphomas. There is positive correlation between FOXO4 expression levels and poor prognosis in these cancers (19). High level of FOXO6 expression has been correlated with poor prognosis and disease progression in gastric cancer (20). Thus, the role of FOXO in cancer cannot be simply determined as a tumor suppressor, and further elucidation of the molecular mechanisms that regulate the roles of FOXO proteins in cancer is needed.

FOXO1 is regulated by various posttranslational modifications such as phosphorylation, methylation, and acetylation (21). Among these modifications, we focused on phosphorylation. An important regulatory mechanism of FOXO is reversible phosphorylation at three sites, namely Thr24, Ser256, and Ser319, catalyzed by AKT (1). Phosphorylated FOXO1 localizes to the cytoplasm and is degraded by the proteasome *via* SKP2-dependent ubiquitination (22). The phosphorylated state of FOXO1, therefore, plays an important role in the physiological functions of FOXO1. However, the role of phosphatase that might dephosphorylate FOXO1 has not been fully investigated.

The serine/threonine phosphatase family consists of PP1 and PP2A, Ca^2+^-dependent PP2B (calcineurin), Mg^2+^-dependent PP2C, PP2A-like phosphatases PP4 (PPX), PP6, PP5, and PP7 (23). Among these, PP2A has been reported to dephosphorylate Thr24 and Ser256 in FOXO1. Phosphorylated FOXO1 promotes transcription of apoptosis-related factors (24). In this study, we focused on PP2B (calcineurin) as a novel phosphatase involved in the regulation of FOXO1 expression. Calcineurin is a protein phosphatase regulated by calcium and calmodulin and is a known target of immunosuppressants, such as cyclosporine and tacrolimus. We have previously reported that calcineurin regulates cell proliferation by promoting the stability of Cyclin D1 (25), ERα (26), EGFR (27), c-Myc (28), and NFATc (29).

Furthermore, we focused on p53 as a target of FOXO1. p53 is a crucial tumor suppressor, exhibiting a wide array of functions encompassing regulation of cell cycle arrest and initiation of apoptosis. p53-induced cell cycle arrest is mediated by the CDK inhibitor p21 (also called as WAF1, CIP1), the first identified transcriptional target of p53 (30). The key p53 regulator is ubiquitinating enzyme MDM2. MDM2 binds to and ubiquitinates p53, thereby promoting its degradation by the 26S proteasome and maintaining p53 at low levels. Immediately after the cell is stressed, the binding of MDM2 to p53 is inhibited or altered to prevent MDM2-mediated degradation. As a result, p53 levels increase, leading to cell cycle arrest and apoptosis (31, 32). Recently, FOXO1 was shown to repress transcription of p53 hepatic p53 mRNA directly upon fasting (33). Regulation of p53 by other FOXO proteins has also been reported. FOXO3a localizes p53 to the cytoplasm (34, 35, 36) and FOXO4 interacts with p53 (37), thereby inhibiting its function. In this study, we showed that FOXO1 induced p53 degradation by activating *MDM2* transcription, thereby promoting cancer cell progression. We showed that FOXO1 suppresses p53 *via* a completely different mechanism than FOXO3a and FOXO4.

## Results

### The most frequently mutated site of FOXO1 in cancer is in the region surrounding Thr24

**Figure 1:**
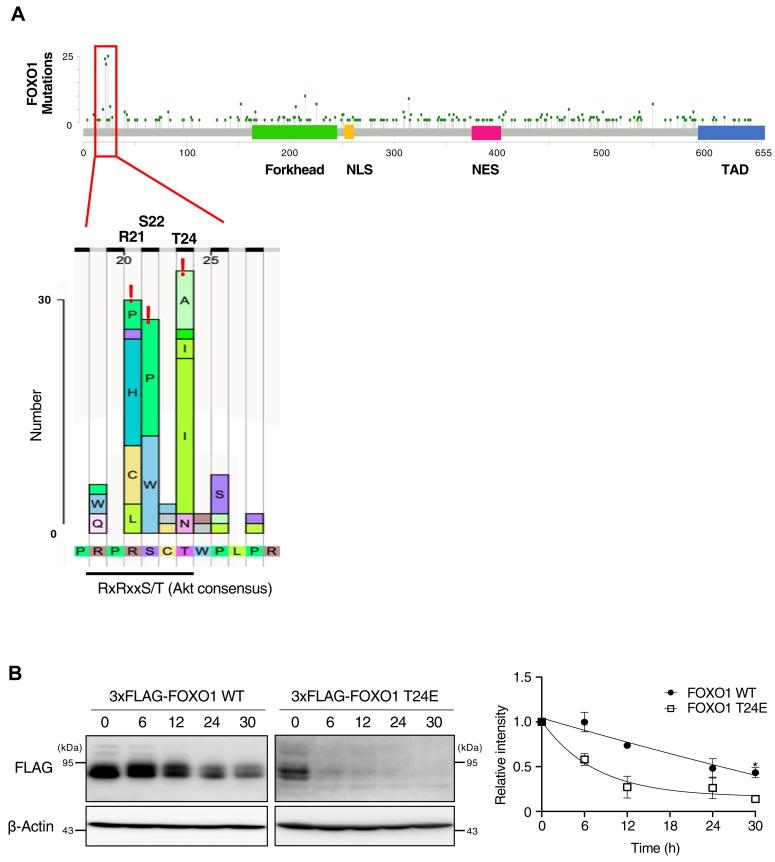
**The most frequently mutated site of FOXO1 in cancer is in the region surrounding Thr24.***A*, the distribution and frequency of missense somatic mutations in FOXO1 in all cancers were obtained from the COSMIC database and visualized using the Mutation Mapper. *B*, MCF7 cells were transiently transfected with expression vectors for FLAG-tagged WT and T24E mutant forms of FOXO1 and incubated with cycloheximide (CHX) for the indicated times, following which cell lysates were subjected to immunoblotting with the indicated antibodies. ∗*p* < 0.05 by two-tailed Student’s *t* test.

To clarify the role of FOXO1 in cancer cells, we first examined the location and frequency of missense mutations in all cancer somatic mutation data obtained from COSMIC (38). We found that mutations were concentrated in and around Thr24 (Fig. 1*A*). It is one of the three amino acids phosphorylated by AKT, and T24I/A and mutants with 3 A substitutions are known to be functionally active (12, 13). The consensus sequence for phosphorylation by AKT is RxRxxS/T (39). The top three amino acids with high mutation frequencies in this analysis, namely Arg21, Ser22, and Thr24, were included in the consensus sequence for AKT. As these mutations are expected to interfere with the phosphorylation of Thr24 by AKT, FOXO1 may be dephosphorylated, activated, and stabilized in cancer cells. Indeed, we found that T24I or T24A mutations likely affected FOXO1 function (Table 1). We examined the stability of the FOXO1 mutant in which Thr24 was replaced with glutamic acid (T24E), using a cycloheximide chase assay. T24I/A was not used in this assay because its transcriptional activity was quite high (12, 13) and cells expressing these mutants had extremely poor proliferation. FOXO1-T24E was destabilized compared to WT (Fig. 1*B*), suggesting that the unphosphorylated form of FOXO1 observed in cancer cells was more stable than WT.

**Table 1:** Functional prediction and annotation of FOXO1 T24I/A mutations by dbNSFP v4

Gene	Protein change	SIFT44G		Polyphen2 HDIV		Polyphen2 HVAR		Mutation taster		Mutation assessor		FATHMM-XF	
		Score	Prediction	Score	Prediction	Score	Prediction	Score	Prediction	Score	Prediction	Score	Prediction
FOXO1	p.T24I	0	Deleterious	0.998	Probably damaging	0.99	Probably damaging	0.99999	Disease causing	2.9	Medium	0.620447	Deleterious
	p.T24A	0	Deleterious	0.993	Probably damaging	0.968	Probably damaging	0.99983	Disease causing	2.9	Medium	0.591841	Deleterious

		**MutPred Top5 features**											
		Gain of catalytic residue at P28 (*p* = 0.0013); loss of phosphorylation at T24 (*p* = 0.0208)											
		Loss of glycosylation at T24 (*p* = 0.1159); loss of sheet (*p* = 0.437); loss of methylation at R19 (*p* = 0.4407)											
		Gain of catalytic residue at P28 (*p* = 0.0029); loss of phosphorylation at T24 (*p* = 0.0208)											
		Loss of glycosylation at T24 (*p* = 0.1159); gain of methylation at R19 (*p* = 0.3967); loss of stability (*p* = 0.5159)											

Somatic mutation data were obtained from COSMIC. Functional prediction and annotation of missense mutations were performed using dbNSFP v4.

### The protein expression of FOXO1 is positively correlated with that of calcineurin

**Figure 2:**
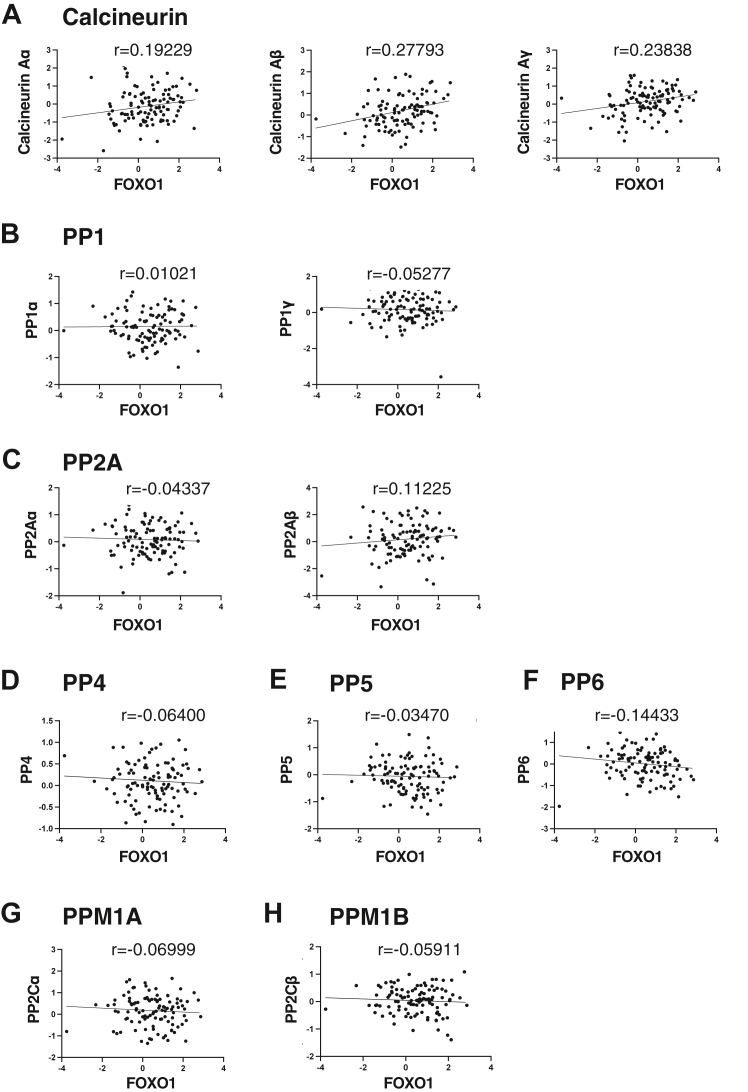
**The protein expression of FOXO1 is positively correlated with that of calcineurin among phosphatases.***A*–*H*, Data from a large proteomic analysis of breast cancer patients (Krug *et al.* 2020. Cell) were obtained, and Pearson correlation coefficients were analyzed between FOXO1 and protein phosphatases, Calcineurin (*A*), PP1 (*B*), PP2A (*C*), PP4 (*D*), PP5 (*E*), PP6 (f), PPM1A (*G*), and PPM1B (*H*).

Since mutations in FOXO1 are often found at AKT phosphorylation sites in cancer, we focused on protein phosphatases as new factors that might modulate FOXO1 phosphorylation. Since Thr24 phosphorylation is an indicator of ubiquitination and degradation by Skp2 (22), phosphatases that dephosphorylate Thr24 may be positively correlated with FOXO1. To search for protein phosphatases that dephosphorylate FOXO1, we obtained data from a large proteomic analysis of breast cancer patients (40) and calculated the Pearson correlation coefficients between FOXO1 and protein phosphatases. These protein phosphatases include the PPP family, which dephosphorylates serine/threonine, and PPM family, which dephosphorylates tyrosine (23). We found that three isoforms of calcineurin catalytic subunit, PPP3CA, CB, and CC are positively correlated with FOXO1 (Fig. 2). This indicated that calcineurin may regulate FOXO1 protein expression.

### Calcineurin is required for the expression of FOXO1

**Figure 3:**
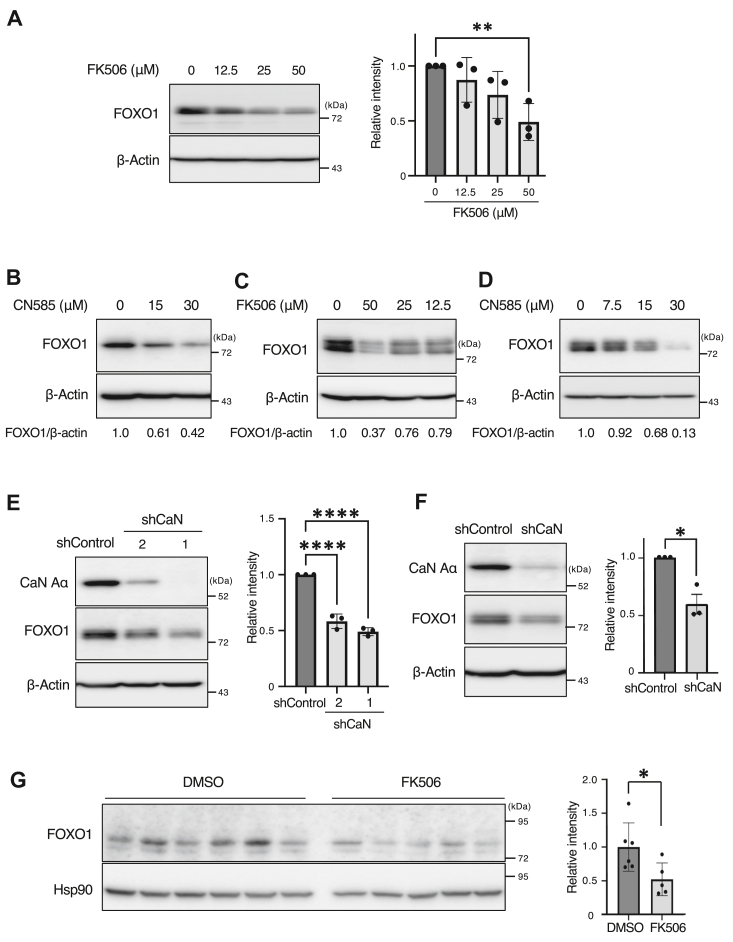
**Calcineurin is required for the expression of FOXO1.***A*, MCF7 cells were treated with the indicated concentrations of FK506 for 24 h. The total cell lysates were prepared and immunoblotted. Representative immunoblots and the relative band intensity (FOXO1/β-actin) determined as the mean ± SEM from three independent experiments are shown. Each value was tested using one-way ANOVA, followed by Dunnett’s test. ∗∗*p* < 0.01, ∗∗∗∗*p* < 0.0001. *B*, MCF7 cells were treated with the indicated concentrations of CN585 for 8 h, following which total cell lysates were prepared and analyzed as described in (*A*). *C* and *D*, HT29 cells were treated with the indicated concentrations of FK506 for 24 h (*C*) or CN585 for 24 h (*D*), after which total cell lysates were prepared and analyzed as in (*A*). *E* and *F*, lentivirus-infected MCF7 (*E*) or HT29 (*F*) cells treated with calcineurin Aα (shCaN Aα) shRNA or luciferase (shControl) shRNAs. Two different calcineurin Aα shRNAs (1 and 2) were used in this study. Cell lysates were prepared and analyzed as described in (*A*). Quantitative data are presented as mean ± SEM from three independent experiments. Each value was tested using two-tailed Student's *t* test. ∗*p* < 0.05, ∗∗∗∗*p* < 0.0001. *G*, HT29 cells were transplanted into the flanks of NOD/Shi-SCID mice. Xenograft tumors after treatment on day 21 were subjected to immunoblotting. Individual lanes represent each mouse. Error bars represent the mean ± SEM. Each value was tested using two-tailed Student's *t* test.

To examine the possibility that calcineurin is involved in the expression of FOXO1, we first measured the effect of FK506, a calcineurin inhibitor, on the expression of FOXO1. Immunoblot analysis revealed that FOXO1 expression in MCF7 cells was decreased by FK506 treatment in a concentration-dependent manner (Fig. 3*A*). To investigate the involvement of phosphatase activity in FOXO1 expression, cells were treated with CN 585, which specifically inhibits calcineurin phosphatase activity (41, 42, 43, 44). We observed a concentration-dependent decrease in FOXO1 expression in MCF7 cells following CN585 treatment (Fig. 3*B*). Furthermore, inhibition of calcineurin by FK506 or CN585 also resulted in a decrease in the protein levels of FOXO1 in HT29 (Fig. 3, *C* and *D*), 22Rv1 (Fig. S1*A*), and LNCaP cells (Fig. S1*B*). To test the specificity of calcineurin inhibition on FOXO1 expression, calcineurin Aα targeting by two independent shRNAs was evaluated. Calcineurin knockdown reduced the protein level of FOXO1 in MCF7 cells (Fig. 3*E*) and HT29 cells (Fig. 3*F*), compared to the controls.

Since PP2A is known to dephosphorylate FOXO1 (24), we examined the contribution of PP2A in FOXO1 stability. In PP2A-depleted cells, phosphorylation of FOXO1 at Thr24 was increased, and FOXO1 protein was decreased (Fig. S1*C*), suggesting that FOXO1 dephosphorylation mediated by PP2A also promotes its stabilization.

Next, we examined FOXO1 expression after FK506 administration *in vivo*, using a mouse xenograft model. HT29 cells were subcutaneously implanted into the flanks of the NOD/Shi-SCID mice (28). FK506 was injected daily when the tumor volume reached approximately 150 mm^3^. After 3 weeks of treatment, proteins were extracted from the collected tumors and FOXO1 expression was examined. The results showed that FOXO1 expression decreased in tumors treated with FK506 (Fig. 3*G*). These results suggest that calcineurin inhibition is effective to suppress tumor progression.

### Calcineurin inhibition decreases FOXO1 in a Skp2-dependent manner

**Figure 4:**
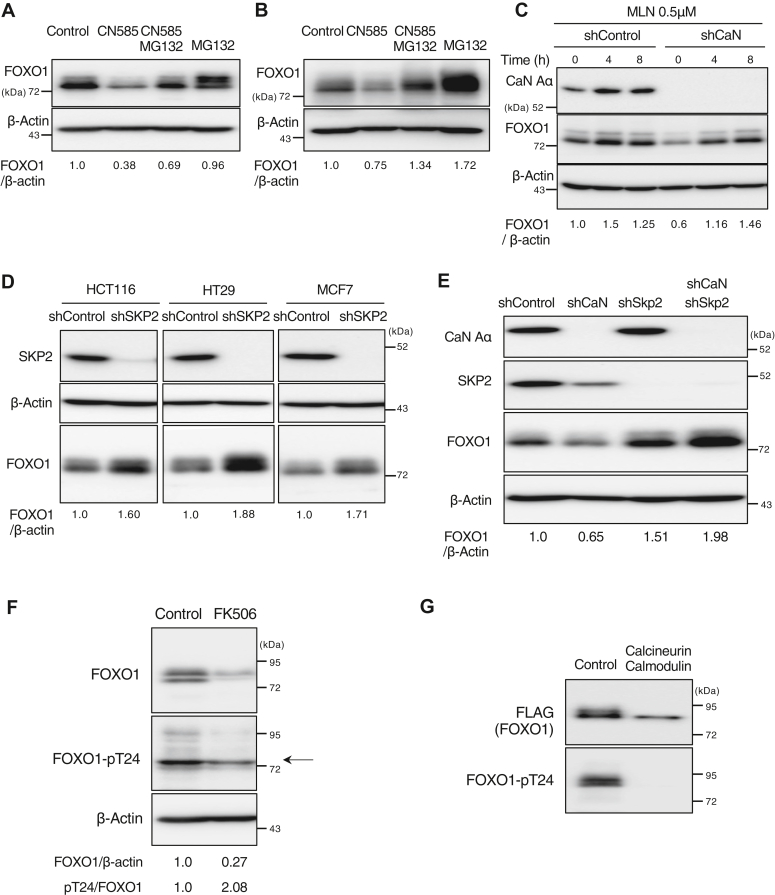
**Calcineurin inhibition decreases FOXO1 in Skp2-dependent manner.***A* and *B*, MCF7 cells treated with CN585 (25 μM) or with without MG132 (10 μM) for 12 h (*A*) and HT29 cells treated with CN585 (25 μM) with or without MG132 (10 μM) for 24 h (*B*) were subjected to immunoblotting. *C*, MCF7 cells expressing calcineurin A–α (shCaN Aα) or luciferase (shControl) shRNAs by exposure to Dox were treated with MLN4924 (0.5 μM) for the indicated times and were subjected to immunoblotting. *D*, HCT116/HT29/MCF7 cells expressing SKP2 shRNAs (shSKP2) or luciferase (shControl) shRNAs were collected, and total cell lysates were analyzed by immunoblotting. *E*, MCF7 cells expressing the indicated shRNAs were collected, and total cell lysates were analyzed by immunoblotting. *F*, HT29 cells treated with FK506 (50 μM) or with without MG132 (10 μM) for 24 h were subjected to immunoblotting. The relative FOXO1-phosphorylated T24/FOXO1 band intensity ratio was determined. *G*, phosphatase assay was performed with immunoprecipitated FLAG-tagged FOXO1, with or without recombinant calcineurin and calmodulin. The relative FOXO1 phosphorylated Thr24/FLAG band intensity ratio was determined.

To determine whether the decrease in FOXO1 induced by calcineurin inhibition was due to proteolysis *via* the ubiquitin-proteasome pathway, we evaluated the effect of the proteasome inhibitor MG132. FOXO1 decrease associated with CN585 treatment suppressed by MG132 in MCF7 (Fig. 4*A*) and HT29 cells (Fig. 4*B*), indicating that calcineurin inhibition promotes degradation of FOXO1. Next, when ubiquitin-proteasome degradation was inhibited in MCF7 cells with calcineurin knockdown using MLN4924, a drug that inhibits the NEDD8-activating enzyme, the decrease in FOXO1 protein due to calcineurin knockdown was suppressed (Fig. 4*C*). To confirm the relationship between the ubiquitinating enzyme SKP2 and FOXO1, depletion of SKP2 in HCT116/HT29/MCF7 cells increased FOXO1 protein levels in all cells, as expected (Fig. 4*D*). In MCF7 cells, calcineurin knockdown suppressed the decrease in FOXO1 protein level (Fig. 4*E*). These findings indicated that calcineurin promotes FOXO1 stability by inhibiting the ubiquitin–proteasome pathway.

### Calcineurin dephosphorylates FOXO1 at Thr24

To examine the importance of calcineurin on FOXO1 phosphorylation, we examined the phosphorylation state of Thr24 in calcineurin-inhibited HT29 cells treated with FK506, in the presence of MG132. The results showed that calcineurin inhibition increased Thr24 phosphorylation, indicating that Thr24 dephosphorylation was inhibited (Fig. 4*F*). Next, we tested whether calcineurin induces the dephosphorylation of FOXO1 at Thr24 using an *in vitro* phosphatase assay and found that calcineurin dephosphorylates Thr24 (Fig. 4*G*), one of the AKT phosphorylation sites.

### FOXO1 induces p53 degradation by promoting *MDM2* transcription

**Figure 5:**
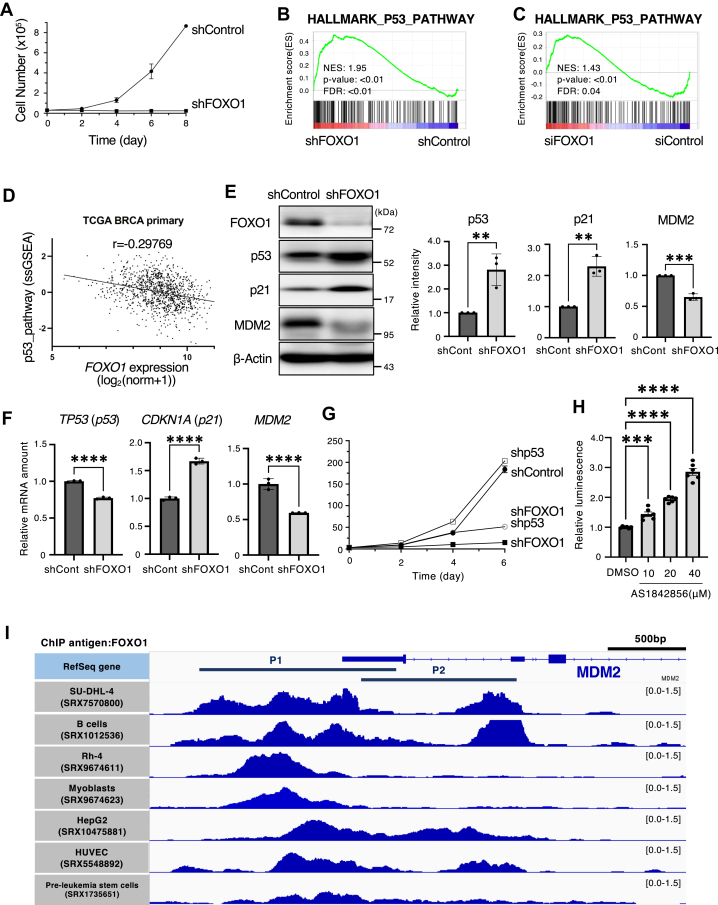
**Growth inhibition by FOXO1 depletion is partially dependent on p53.***A*, growth curves of MCF7 cells expressing FOXO1 (shFOXO1) or luciferase (shControl) shRNA after exposure to Dox. Cell numbers were counted every 2 days. *B*, the enrichment plot of HALLMARK_p53_Pathway in MCF7 cells treated with FOXO1 (shFOXO1) shRNA or luciferase (shControl) shRNAs was generated using GSEA software. Three biological replicates were analyzed. *C*, the enrichment plot of HALLMARK_p53_Pathway in the FOXO1-depletion HUVEC cells (GSE116033) compared with the control was generated using GSEA software. *D*, the dataset of TCGA BRCA primary cancer from UCSC Xena(https://xenabrowser.net/) were obtained and Pearson correlation coefficients were calculated. *E*, MCF7 cells expressing the indicated shRNAs were collected, and total cell lysates were analyzed by immunoblotting. Representative immunoblots and the relative band intensity (p53, p21, MDM2/β-actin) determined as the mean ± SEM from three independent experiments are shown. Each value was tested using one-way ANOVA, followed by Dunnett’s test. ∗∗*p* < 0.01, ∗∗∗∗*p* < 0.0001. *F*, total RNA isolated from MCF7 cells expressing the indicated shRNA was subjected to RT-quantitative polymerase chain reaction to analyze the indicated mRNA. RT-quantitative polymerase chain reaction data are presented as the mean ± SEM from three independent experiments. Each value was tested using two-tailed Student's *t* test. ∗∗∗∗*p* < 0.0001. *G*, growth curves of MCF7 cells expressing FOXO1 (shFOXO1), TP53 (shp53), or luciferase (shControl) shRNA after exposure to Dox. Cell numbers were counted every 2 days using a cell counter. *H*, MCF7 cells were transiently transfected with the p53-RE-Nluc reporter plasmid. After 24 h, cells were treated with the indicated concentrations of AS1842856. The luciferase activity was measured after 24 h. Data are expressed as mean ± SEM. Each value was tested using one-way ANOVA, followed by Dunnett’s test. ∗∗∗*p* < 0.001, ∗∗∗∗*p* < 0.0001. *I*, bigwig formatted ChIP-Seq data of chromatin immunoprecipitated with FOXO1 antibody in several cell lines were obtained *via* ChIP-Atlas (https://chip-atlas.org/). The binding peaks of FOXO1 for each cell lines were displayed using IGV, and two promoter regions of *MDM2* were added to the corresponding locations (55). FDR, false discovery rate; IGV, integrative genomics viewer; NES, normalized enrichment score; ssGSEA, single sample gene set enrichment analysis.

Although FOXO1 is known as a tumor suppressor, it also contributes to malignant transformation in lymphomas (11). Therefore, we evaluated the effects of FOXO on cancer cell proliferation. When FOXO1 was depleted using an shRNA in the MCF7 human breast cancer cell line (Fig. 5*A*) and HT29 human colon cancer cell line (Fig. S2*A*), cell growth was markedly inhibited. To determine why cell proliferation is inhibited by FOXO1 depletion, RNA-seq analysis of FOXO1-depleted MCF7 cells was performed. The effects of shRNAs were clearly observed by hierarchical clustering and principal component analysis using RNA-seq data (Fig. S3, *A* and *B*). Gene set enrichment analysis revealed that p53 pathway is activated in FOXO1-depleted cells (Figs. 5*B* and S3*C*). Similar result was also observed in FOXO1-depleted HUVEC cells (GSE116033) (Fig. 5*C*). In also breast cancer patient samples on TCGA BRCA, the activation score of the p53 pathway predicted by PARADIGM was negatively correlated with the expression level of *FOXO1* mRNA (Fig. 5*D*). Consistent with these results, we found that FOXO1 depletion increased the protein amount of p53 and its target, p21 (Figs. 5*E* and S2*B*). In addition, transcription of these genes was reduced in FOXO1-depleted cells (Fig. 5*F*). To evaluate the activated p53 in FOXO1-depleted cells, we depleted FOXO1 and p53 alone or together using shRNA in MCF7 cells. We found that the decrease in proliferation caused by FOXO1 depletion was partially restored by p53 depletion (Fig. 5*G*).

To examine the changes in p53 transcriptional activity upon FOXO1 inhibition, we used cells treated with AS1842856, a FOXO1 inhibitor, in a dual-luciferase assay. p53 transcriptional activity increased in a concentration-dependent manner, indicating that FOXO1 inhibition increased transcriptional activity of p53 (Fig. 5*H*). We hypothesized that the increase in p53 upon FOXO1 depletion might be due to a decrease in MDM2, a representative E3 ligase of p53. Indeed, MDM2 protein levels were reduced when FOXO1 was depleted (Fig. 5*E*). However, in the prostate cancer cell lines 22Rv1 and LNCaP, where FOXO1 has been reported to act in a tumor-suppressive manner (5), depletion of FOXO1 did not alter MDM2 and p53 expression (Fig. S2, *C* and *D*). This suggests that the regulation of MDM2 and p53 by FOXO1 is cell type–dependent.

*MDM2* mRNA levels also decreased (Fig. 5*F*), suggesting that FOXO1 may regulate *MDM2* gene expression at the transcriptional level. Therefore, we examined whether FOXO1 binds to the promoter region of the *MDM2* gene using publicly available ChIP-Seq data obtained *via* ChIP-Atlas. Binding peaks of FOXO1 were detected in the promoter region of *MDM2* (Fig. 5*I*), and binding peaks to the *MDM2* promoter region was observed to be higher than binding peaks to the *CDKN1A* (encodes p21) and *CDKN1B* (encodes p27) promoter region in several cell lines (Fig. S4, *A* and *B*), suggesting that FOXO1 binds directly to *MDM2* genomic locus and promotes its expression as a transcription factor. These findings suggested that FOXO1 induces p53 degradation by promoting *MDM2* transcription.

## Discussion

The FOXO family of transcription factors, consisting of four members—FOXO1, FOXO3, FOXO4, and FOXO6—play a role in transcriptional activation of gluconeogenesis (2, 3), the cyclin-dependent kinase inhibitor, such as p27 (4, 5) and p21 (6), and several proapoptotic genes (7). This leads to cell cycle arrest in the G1 phase or induces apoptosis. FOXO proteins function as tumor suppressors by inhibiting cell cycle progression, angiogenesis, and metastasis, and by promoting apoptosis. However, other reports have suggested that FOXOs can also facilitate tumor progression, indicating that their functions in malignant tumors can vary depending on the circumstances. FOXOs, therefore, may influence various aspects of cancer onset and progression by regulating the expression of downstream target genes.

In this study, we show that FOXO1 promotes cancer cell proliferation by repressing p53 through activating the transcription of *MDM2* (Fig. S5). We focused on the mutation of Thr24 of FOXO1, a residue that is frequently mutated in cancer. AKT mediated phosphorylation of FOXO1 at three sites, Thr24, Ser256, and Ser319, and promotes the binding of nuclear 14-3-3 protein, leading to FOXO export out of the nucleus (1). Phosphorylation of these residues increase the binding of Skp2, an F-box protein that is a part of the SCF (Skp1-Cullin-F-box protein) ubiquitin ligase complex, causing FOXO1 degradation. Besides phosphorylation, FOXO is regulated by multiple posttranslational modifications. Acetylation of Foxo1 is known to reduce its ability to interact with target genomic DNA and promote phosphorylation of Ser-253 of Foxo1 (45). Arginine methylation of FOXO1 inhibits its phosphorylation by Akt, Thus, posttranslational modifications of FOXO1 primarily determine subcellular localization, transcriptional activity, and protein stability (21).

We identified calcineurin as possible regulator to stabilize the FOXO1 protein by analyzing Pearson correlation coefficients between various protein phosphatases and FOXO1. We demonstrated that calcineurin dephosphorylates Thr24 and increase the stability of FOXO1. Dephosphorylated FOXO1 at Akt-mediated phosphorylation sites escapes degradation by the ubiquitin-proteasome system and becomes highly activated (1). This study revealed that calcineurin acts upstream of FOXO1 and regulates MDM2 and p53 expression by stabilizing FOXO1. We previously reported that calcineurin is highly expressed in cancer (26) and that calcineurin also stabilizes and activates factors promoting cancer cell proliferation, such as Cyclin D1 (25), estrogen receptor ERα (26), and the oncogene c-Myc (28).

Given that cancer cell proliferation was markedly decreased in FOXO1-depleted cells, we performed RNA-seq analysis and found that p53 pathway was activated in these cells. p53 is an extremely important tumor suppressor controlled by mainly MDM2, an E3 ubiquitin ligase. FOXO1-depletion showed decreased expression of *MDM2* mRNA as well as increased p53 protein levels. Analysis of publicly available ChIP-Seq data revealed that FOXO1 binds to the promoter region of the *MDM2* gene. This suggests that FOXO1 upregulates MDM2 by activating *MDM2* transcription, leading to p53 degradation and thereby promoting cancer cell proliferation. Transcriptional activation of p21 by FOXO1 has been reported to occur upon TGF-β stimulation (6). However, we found that knockdown of FOXO1 activated p53 protein, thereby inducing transcription of *CDKN1A*. Analysis of public ChIP-seq data also revealed that FOXO1 signal was hardly detected to the promoter region of *CDKN1A* (Fig. S4*A*). This suggests that in the absence of TGF-β stimulation, FOXO1 represses p21 expression *via* repression of p53.

It has also been reported that FOXO3a binds directly to p53 and localizes p53 to the cytoplasm (34, 35, 36). Thus, FOXO protein functions to repress p53 by promoting *MDM2* transcription *via* FOXO1 and by mediating the cytoplasmic localization of p53 *via* FOXO3a. These results strongly suggest that the FOXO protein family, which is classically considered to be tumor suppressors, may also have paradoxical roles in promoting cancer.

A tumor-suppressive role for FOXO1 has been shown in prostate cancer cell lines (5). FOXO1 depletion in these cells, however, did not alter the protein expression of MDM2 and p53 (Fig. S2, C and D). This indicates that the involvement of FOXO1 in MDM2 expression may be cell type–dependent and that transcription factors other than FOXO1 may regulate MDM2 expression in prostate cancer cells. We, therefore, speculate that FOXO1 may act in a cancer-promoting manner in certain cancer cell types by activating MDM2 transcription. However, in some other cancer cell types, in which FOXO1 does not regulate MDM2 transcription but instead activates *CDKN1B* transcription, FOXO1 may paradoxically act in a cancer-suppressing manner.

## Experimental procedures

### Cell culture and reagents

MCF7 (HTB-22; ATCC) and HEK293T (632180; Takara) cells were cultured in Dulbecco's modified Eagle's medium (044-29765; WAKO) supplemented with 10% fetal bovine serum (FBS) (173012; Sigma) and an antibiotic-antimycotic solution (15240062; Thermo Fisher Scientific). HT29 (HTB-38; ATCC) and HCT116 (CCL-247; ATCC) cells were cultured in McCoy's 5A medium (16600-082; Gibco) containing 10% FBS and antibiotic-antimycotic solution. 22Rv1 (CRL-2505; ATCC) and LNCaP (CRL-1740; ATCC) cells were cultured in RPMI medium (187-02705; WAKO) containing 10% FBS and antibiotic-antimycotic solution. All cells were cultured at 37 °C under 5% CO_2_. The cells were treated with FK506 (S500313; Selleckchem), MG132 (S261917; Selleckchem), and CN585 (207003; Merck). FK506 was used at concentrations of 50 μM, 25 μM, 12.5 μM or 6.25 μM, MG132 at 10 μM, and CN585 at 30 μM, 25 μM, 15 μM, or 7.5 μM. Cycloheximide (037-20991; FUJIFILM) was used at a concentration of 50 μg/ml. MLN4924 (S7109; Selleckchem) was used at a concentration of 0.5 μM. Okadaic acid (152-03271; FUJIFILM) was used at concentrations of 50 μM or 25 μM. AS1842856 (HY-100596; MedChemExpress) was used at a concentration of 40 μM, 20 μM, or 10 μM.

### RNA-seq analysis

MCF7 cells expressing shControl or shFOXO1 were cultured in the presence of Dox for 3 days and then subjected to total RNA extraction with the use of a RNeasy Plus Mini Kit (74134; Qiagen). The RNA integrity number was measured with an Agilent 2100 Bioanalyzer to evaluate RNA quality. Poly(A)+ RNA was then isolated with the use of an NEBNext Poly (A) mRNA Magnetic Isolation Module (E7490; New England BioLabs [NEB]), and a cDNA library was prepared with an NEBNext Ultra II Directional RNA Library Prep Kit for Illumina (E7760; NEB) and sequenced with the Illumina NovaSeq 6000 system (20012850; Illumina). Publicly available RNA-seq data for siControl and siFOXO1 human umbilical vein endothelial cells were obtained from Gene Expression Omnibus under accession number GSE116033 (46). Raw counts were obtained using RaNA-seq (47). Raw counts were subjected to DEBrowser v1.28.0 (48) for low reads (genes with a maximum count of less than 10 in each sample) removal and quality control of RNA-seq analysis. Filtered counts were normalized using edgeR v3.40.2 (49, 50). Gene Set Enrichment Analysis with normalized counts was performed using GSEA software v4.20 (51, 52).

### ChIP-seq analysis

Bigwig formatted ChIP-Seq data of chromatin immunoprecipitated with FOXO1 antibody in several cell lines were obtained *via* ChIP-Atlas (https://chip-atlas.org/) (53, 54) under accession number SRX7570800, SRX1012536, SRX9674611, SRX9674623, SRX10475881, SRX5548892, and SRX1735651. Binding peaks in the promoter region of the gene were visualized by Integrative Genomics Viewer (https://software.broadinstitute.org/software/igv/home).

Promoter regions are indicated corresponding to previously reported (55, 56, 57, 58).

### Construction of expression vectors and mutagenesis

For FOXO1 overexpression in cells, the human FOXO1 corresponding ORF (NM_002015.4) was cloned into the N-terminally 3× FLAG-tagged pcDNA3 vector. The FOXO1 mutant T24E was generated using the NEBuilder HiFi DNA Assembly Master Mix (E2621; NEB). The oligo with Thr24(ACC) replaced by Glu (GAA) was diluted in TE buffer (10 mM Tris–HCL, 0.1 mM EDTA, and 50 mM NaCL) and incubated with linearized pcDNA3 FLAG-FOXO1 WT and NEBuilder HiFi DNA Assembly Master Mix at 50 °C for 1 h to produce the pcDNA3 FLAG-FOXO1 T24E vector.

### Dual luciferase reporter assay

MCF7 cells (1 × 10^4^ cells/well) were cultured in Dulbecco’s modified Eagle’s medium supplemented with 10% FBS in white 96-well plates for 24 h. Cells were transfected with 48 ng pNL[NlucP/p53-RE/Hygro] Vector (CS194102; Promega) and 2 ng pGL4.51[luc2/CMV/Neo] Vector (E1320; Promega) using 150 ng PEI Max (24765-1; Polyscience). Forty-eight hours after transfection, NanoLuc luciferase and firefly luciferase activity were measured using the Nano-Glo Dual-Luciferase Reporter Assay System (N1610; Promega) and a Nivo S microplate reader (PerkinElmer). The luminescence elicited by NanoLuc was normalized to that elicited by firefly luciferase.

### Lentivirus generation and infection

Lentivirus generation and infection were performed as described previously (59). Lentiviruses expressing shControl, shCalcineurin, shFOXO1, shSKP2, or shp53 were generated by the co-transfection of HEK293T cells with psPAX2 and pMD2.G and the respective CS-RfA-ETBsd using PEI Max (24765-1; Polyscience). Infected cells were treated with 10 μg/ml blasticidin (A1113903; Gibco) or 200 μg/ml hygromycin (H3274; Sigma-Aldrich) for 2 days. Doxycycline (Dox, 1 μg/ml, D9891; Sigma-Aldrich) was used to express shRNA. The target sequences for lentivirus-based shRNA are listed in Table S1.

### Transient transfection

HEK293T cells were transfected with pcDNA3 FLAG-FOXO1 WT and pcDNA3 FLAG-FOXO1 T24E mutants using PEI Max (24765-1; Polyscience) transfection reagent.

### Immunoblotting

Immunoblotting was performed as described previously (60). Briefly, collected cells were washed with ice-cold PBS, suspended in sample buffer (10% glycerol, 2% sodium dodecyl sulfate, 0.1% bromophenol blue, 100 μM DTT, and 50 mM Tris–HCl at pH 6.8), and boiled for 5 min. After SDS-PAGE and immunoblotting were completed, images were captured using a ChemiDoc Imaging System (Bio-Rad). The antibodies used in this study are listed in Table S2. The band intensities of the target proteins were quantified using Image Lab software (https://www.bio-rad.com/ja-jp/product/image-lab-software?ID=KRE6P5E8Z) and normalized to that of β-actin unless otherwise indicated.

### RT-quantitative polymerase chain reaction analysis

Total RNA was extracted using ISOGEN II (311-07361; Nippon Gene) as described previously (61) and reverse-transcribed with random primers using the High-Capacity cDNA Reverse Transcription Kit (4368814; ABI). Quantitative polymerase chain reaction was performed using the FastStart Universal SYBR Green Master Mix (11226200; Roche) and a StepOnePlus real-time PCR system (Applied Biosystems). The expression levels were normalized to those of β-Actin RNA. Primer sequences were described in Table S3.

### Phosphatase assay

The phosphatase assay was performed as described previously (62). FLAG-FOXO1 was pulled down using the anti-FLAG M2 affinity gel (A2220; Sigma-Aldrich) and eluted with FLAG peptide (F4799; Sigma-Aldrich) in an assay buffer (20 mM Tris, 10 mM MgCl_2_, 0.1 mM CaCl_2_, 1 μg/ml bovine serum albumin, pH 7.5). FLAG-FOXO1 was incubated with or without recombinant human calcineurin (3160-CA; R&D Systems) and calmodulin (208670; Merck) in the assay buffer and incubated for 2 h at 37 °C.

### Mouse xenograft

Animal experiments were approved by the Yamaguchi University Animal Use Committee. All procedures followed applicable guidelines and regulations. Female NOD/Shi-SCID mice (CLEA Japan Inc) were maintained under specific pathogen-free conditions and fed a sterilized standard diet (CE-2; CLEA Japan Inc). HT29 cells (2 × 10^6^ cells/100 μl PBS per site) were transplanted subcutaneously into the left flank. FK506 (S500313; Selleck Chemicals) treatment was initiated when the tumor size reached 150 mm^3^. Mice were intraperitoneally received 3 mg/kg/day FK506 or vehicle control (corn oil) once daily for 3 weeks. From the dissected mice, tumor fragments were harvested and homogenized in radioimmunoprecipitation assay buffer (50 mM Tris–HCl at pH 7.5, 150 mM NaCl, 1 mM ethylenediaminetetraacetic acid, 10% sodium deoxycholate, 10% SDS, and 1% Triton X-100) supplemented with phosphatase inhibitors and protease inhibitors. The supernatant extracted by sonication was then mixed with sample buffer and boiled for 5 min.

### Mutation plot and functional prediction

Somatic mutation frequency data for cancers were obtained from publicly available COSMIC v96 (released May 31, 2022) (38). These data are not personally identifiable. The distribution and frequency of missense mutations were visualized using MutationMapper (https://www.cbioportal.org/mutation_mapper). Functional prediction and annotation of missense mutations were performed using the dbNSFP v4 (released July 27, 2021) (63).

### Correlation coefficient analysis

The relative protein abundance values were obtained from the following database: https://proteomics.broadapps.org/CPTAC-BRCA2020/ (40). *FOXO1* mRNA expression value and PARADIGM pathway activity score (64) of primary cancer in TCGA BRCA were obtained *via* UCSC Xena (https://xenabrowser.net/) (65). Correlation coefficients were calculated and scatter plots were created using GraphPad Prism version 9 (GraphPad Software Inc).

### Statistical analysis

All the data are representative of at least three independent experiments. All statistical analyses were performed using GraphPad Prism version 9. The data are presented as the mean ± SEM. A paired or unpaired *t* test was used to compare the two groups. To compare three or more groups, one-way ANOVA followed by Dunnett multiple comparison test was performed for multiple comparisons. Statistical significance was set at *p* < 0.05. Significance is indicated by ∗*p* < 0.05, ∗∗*p* < 0.01, ∗∗∗*p* < 0.001, and ∗∗∗∗*p* < 0.0001.

## Data availability

RNA-seq raw reads have been submitted to the DNA Data Bank of Japan Sequence Read Archive (DRA)/The National Center for Biotechnology Information Sequence Read Archive (SRA)/European Bioinformatics Institute Sequence Read Archive (ERA) databases under accession number PRJDB16591. Additional data related to this paper may be requested from the authors.

## Supporting information

This article contains supporting information.

## Conflict of interest

The authors declare that they have no conflicts of interests with the contents of this article.
