# Supplementary material for: FOXO1 promotes cancer cell growth through MDM2-mediated p53 degradation
Source: J Biol Chem. 2024 Mar 21;300(4):107209. doi: 10.1016/j.jbc.2024.107209 (PMC11021968; doi:10.1016/j.jbc.2024.107209)
Supplement: Supporting Figures [file mmc1.pdf]

Fig. S1

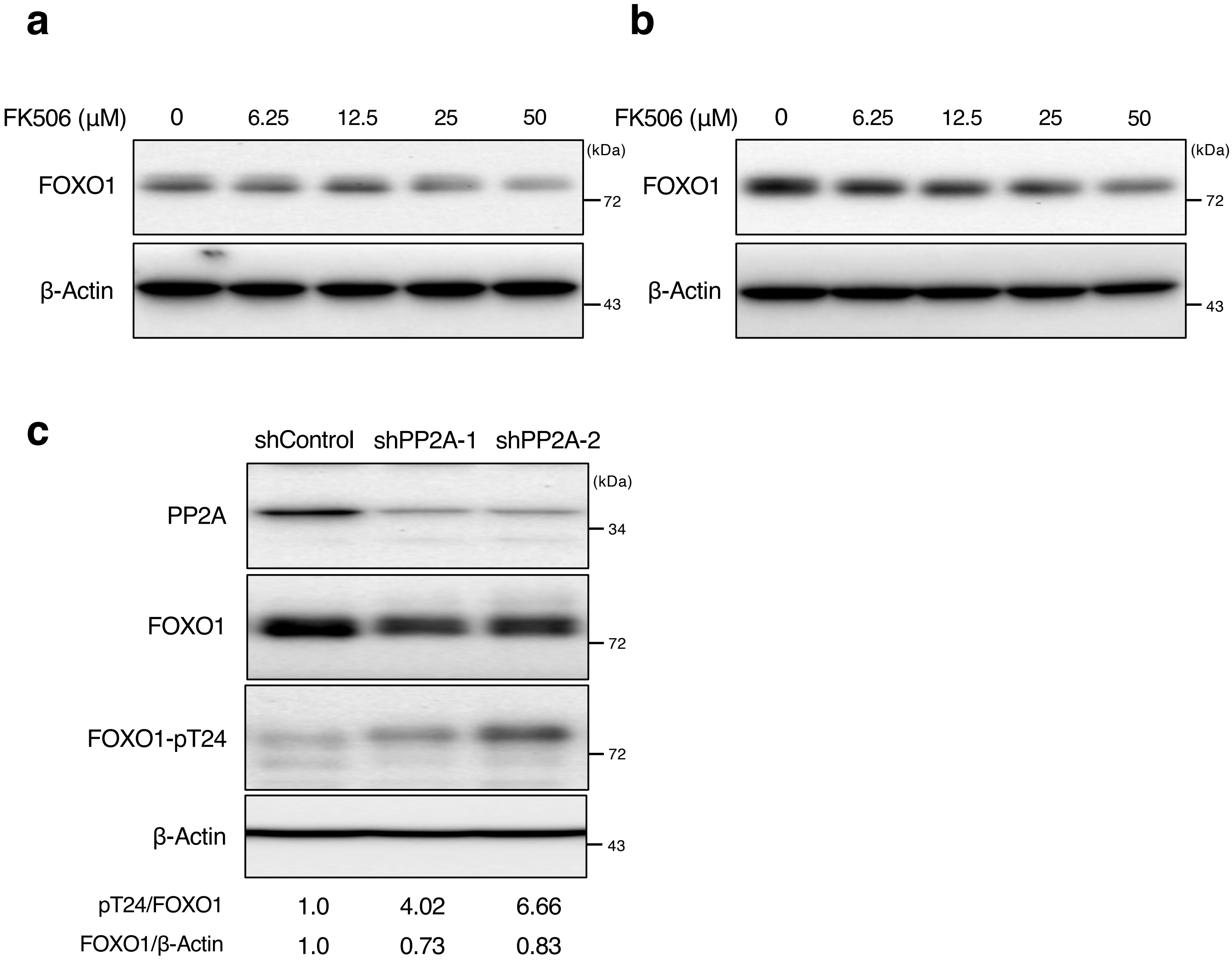

Fig. S1. Effects of phosphatase inhibition on FOXO1 expression

(a)(b) 22Rv1(a) and LNCaP(b) cells were treated with the indicated concentrations of FK506 for 24 h were subjected to immunoblotting.

(c) MCF7 cells expressing the indicated shRNAs were collected, and total cell lysates were analyzed by immunoblotting.

Fig. S2

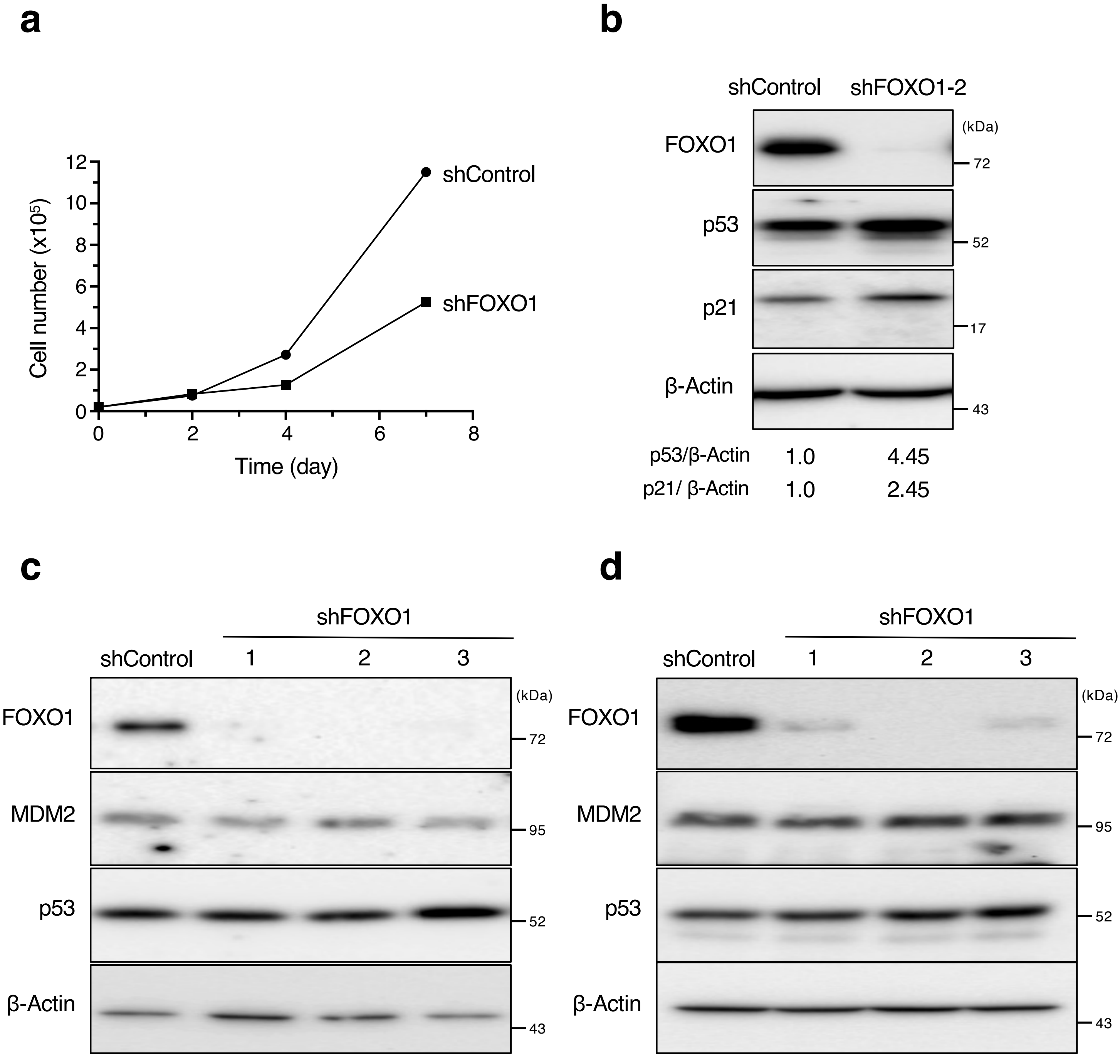

Fig. S2. Effects of FOXO1 depletion on cell proliferation and p53 expression

(a) Growth curves of HT29 cells expressing FOXO1 (shFOXO1) or luciferase (shControl) shRNA after exposure to Dox.

(b) MCF7 cells expressing the indicated shRNAs were collected, and total cell lysates were analyzed by immunoblotting.

(c)(d) 22Rv1(c) and LNCaP(d) cells expressing the indicated shRNAs were collected, and total cell lysates were analyzed by immunoblotting.

Fig. S3

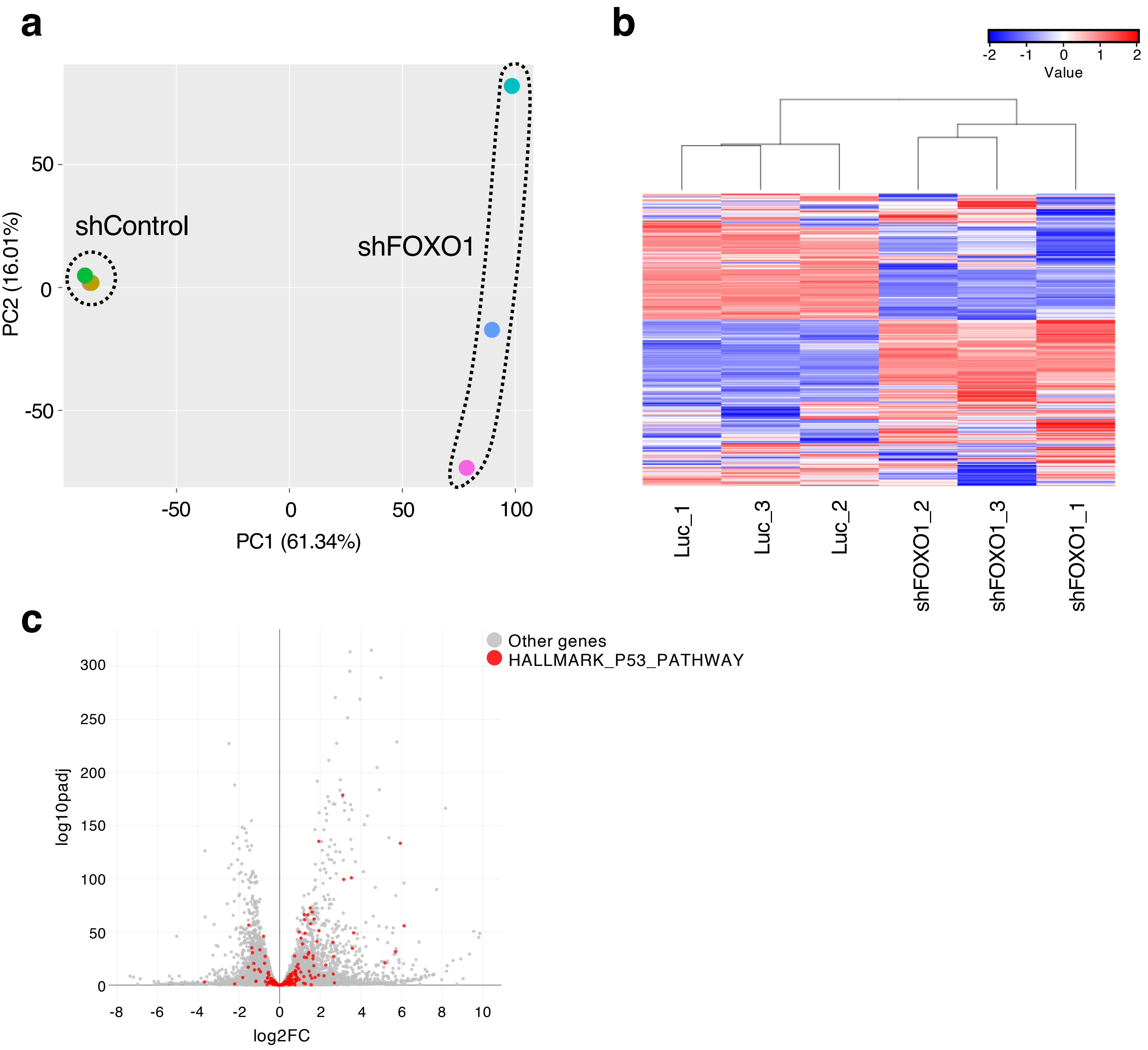

Fig. S3. Quality control for RNA-Seq analysis in MCF7 cells expression shControl and shFOXO1

(a) (b) Counts of all detected genes were analyzed to produce a principal component analysis (PCA) plot (a) and a heatmap (b) for quality control. (c) Volcano plot analysis of all genes detected by the RNA-seq analysis. Red dots indicate genes belonging to the HALLMARK_P53_PATHWAY gene set.

Fig. S4

a

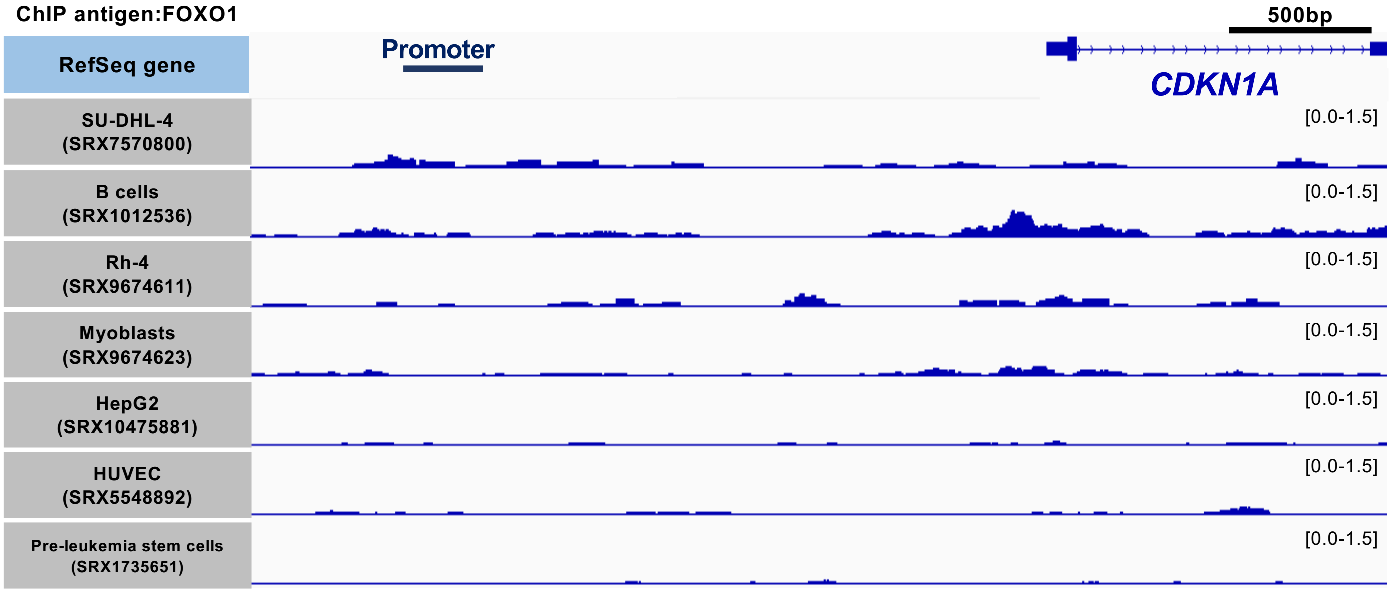

b

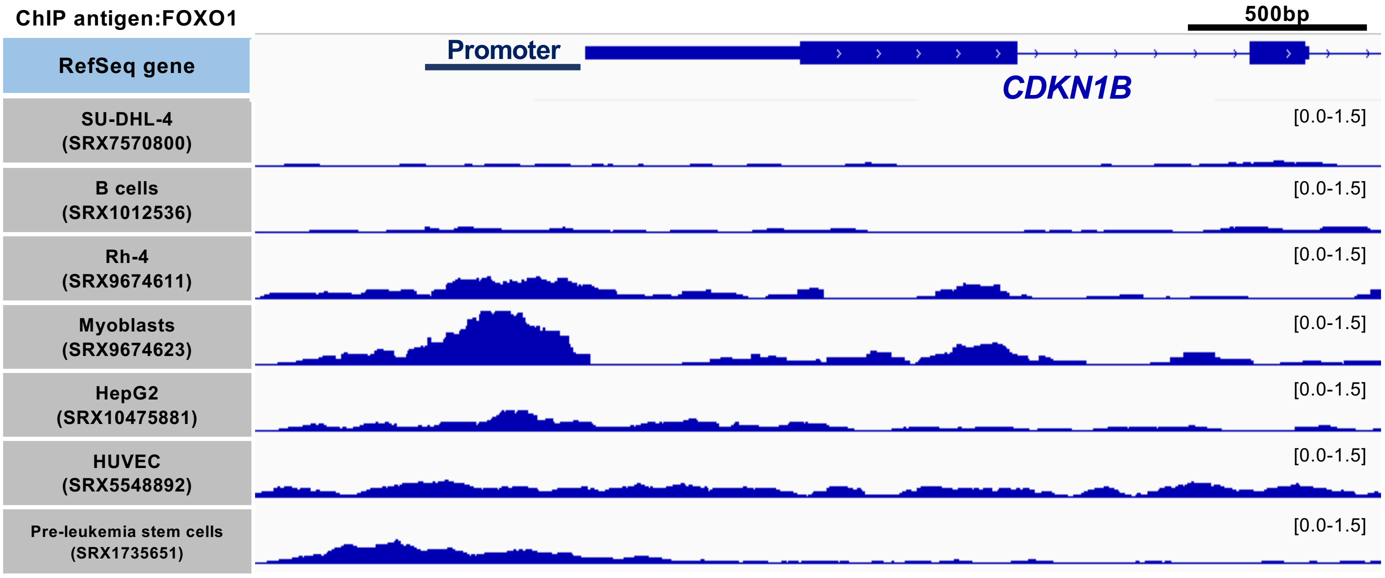

Fig. S4. The binding peaks of FOXO1 to the promoter region of the *CDKN1A* and *CDKN1B*

(a) (b) Bigwig formatted ChIP-Seq data of chromatin immunoprecipitated with FOXO1 antibody in several cell lines were obtained via ChIP-Atlas. The binding peaks of FOXO1 for each cell lines were displayed using IGV, and promoter regions of *CDKN1A/B* were added to the corresponding locations. The data range was adapted to Fig. 5i.

Fig. S5

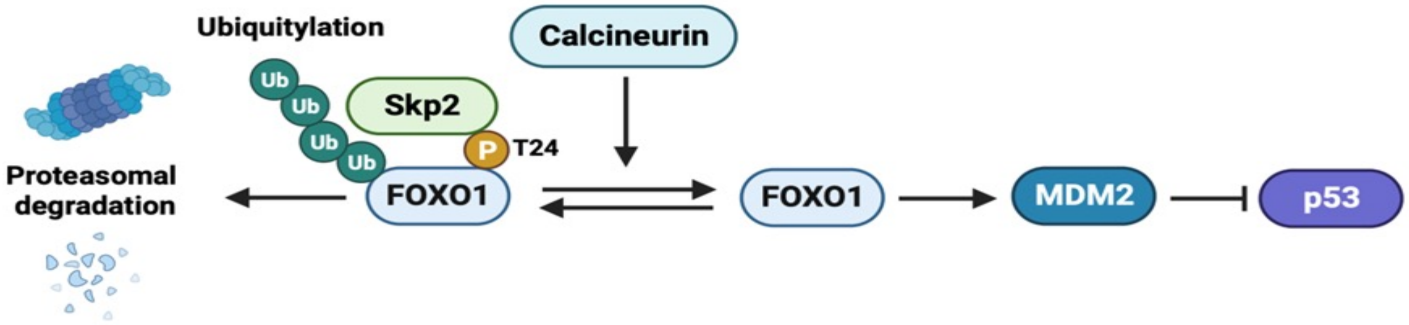

Fig. S5. Model for FOXO1 protein stability and contribution of MDM2-p53 pathway
The phosphorylated form of FOXO1-Thr24 is recognized by Skp2 and promotes its degradation. Dephosphorylation of Thr24 by calcineurin stabilizes FOXO1 and leads to transcriptional activation of *MDM2*, thereby repressing p53.
