# Supplementary material for: FOXO1 promotes cancer cell growth through MDM2-mediated p53 degradation
Source: J Biol Chem. 2024 Mar 21;300(4):107209. doi: 10.1016/j.jbc.2024.107209 (PMC11021968; doi:10.1016/j.jbc.2024.107209)
Supplement: Supporting Tables [file mmc2.docx]

## Supporting information

**Table S1 Sequences of shRNA**

| **Gene** | **Sequence (5’-3’)** |
| --- | --- |
| Luciferase | CGTGCGTGGAATGCTTCGA |
| FOXO1-1 | GCAGCCAGGCATCTCATAACA |
| FOXO1-2 | AAGTTCATTCGTGTGCAGAAT |
| FOXO1-3 | GCTGTTATCAATCTGCTAAT |
| p53 | GACTCCAGTGGTAATCTACT |
| Calcineurin Aα | GCCAAGGGCTTAGACCGAATT |
| Calcineurin Aα-2 | GCAGTTTCTCTGTGTACATGG |
| SKP2 | GGGAGTGACAAAGACTTT |
| PP2A-1 | GATACACTGGATCATATCA |
| PP2A-2 | CTGCAGATAATAAGATGTA |

**Table S2 Antibodies**

| **Antigen** | **Application** | **Catalog No.** | **Company** |
| --- | --- | --- | --- |
| β-Actin | WB | ab6276 | Abcam |
| HSP90 | WB | sc-13119 | Santa Cruz |
| FOXO1 | WB | cs2880 | Cell Signaling |
| FOXO1-pT24 | WB | cs9464 | Cell Signaling |
| Calcineurin Aα | WB | ab52761 | Abcam |
| PP2A | WB | 07-324 | Millipore |
| SKP2 | WB | cs2652 | Cell Signaling |
| p53 | WB | sc-126 | Santa Cruz |
| p21 | WB | sc-6246 | Santa Cruz |
| MDM2 | WB | GTX100531 | Gene Tex |

**Table S3 Sequences of primer for qPCR**

| **Target Gene** | **Forward primer (5’-3’)** | **Reverse primer (5’-3’)** |
| --- | --- | --- |
| *ACTB* | AGAAAATCTGGCACCACACC | AGAGGCGTACAGGGATAGCA |
| *TP53* | CCTCAGCATCTTATCCGAGTGG | TGGATGGTGGTACAGTCAGAGC |
| *p21*(*CDKN1A*) | TTAGCAGCGGAACAAGGAGT | GCCGAGAGAAAACAGTCCAG |
| *MDM2* | TGTTTGGCGTGCCAAGCTTCTC | CACAGATGTACCTGAGTCCGATG |
